# Protective effect of vasoactive intestinal peptide on bone destruction in the collagen-induced arthritis model of rheumatoid arthritis

**DOI:** 10.1186/ar1779

**Published:** 2005-06-23

**Authors:** Yasmina Juarranz, Catalina Abad, Carmen Martinez, Alicia Arranz, Irene Gutierrez-Cañas, Florencia Rosignoli, Rosa P Gomariz, Javier Leceta

**Affiliations:** 1 Departamento Biología Celular, Facultad de Biología, Universidad Complutense de Madrid, Madrid, Spain; 2 Departamento Biología Celular, Facultad de Medicina, Universidad Complutense de Madrid, Madrid, Spain; 3 Servicio de Reumatología y Unidad de Investigación, Hospital 12 de Octubre, Madrid, Spain

## Abstract

Rheumatoid arthritis (RA) is an autoimmune disease of unknown etiology, characterized by the presence of inflammatory synovitis accompanied by destruction of joint cartilage and bone. Treatment with vasoactive intestinal peptide (VIP) prevents experimental arthritis in animal models by downregulation of both autoimmune and inflammatory components of the disease. The aim of this study was to characterize the protective effect of VIP on bone erosion in collagen-induced arthritis (CIA) in mice. We have studied the expression of different mediators implicated in bone homeostasis, such as inducible nitric oxide synthase (iNOS), cyclooxygenase-2 (COX-2), receptor activator of nuclear factor-κB (RANK), receptor activator of nuclear factor-κB ligand (RANKL), osteoprotegerin (OPG), IL-1, IL-4, IL-6, IL-10, IL-11 and IL-17. Circulating cytokine levels were assessed by ELISA and the local expression of mediators were determined by RT-PCR in mRNA extracts from joints. VIP treatment resulted in decreased levels of circulating IL-6, IL-1β and TNFα, and increased levels of IL-4 and IL-10. CIA-mice treated with VIP presented a decrease in mRNA expression of IL-17, IL-11 in the joints. The ratio of RANKL to OPG decreased drastically in the joint after VIP treatment, which correlated with an increase in levels of circulating OPG in CIA mice treated with VIP. In addition, VIP treatment decreased the expression of mRNA for RANK, iNOS and COX-2. To investigate the molecular mechanisms involved, we tested the activity of NFκB and AP-1, two transcriptional factors closely related to joint erosion, by EMSA in synovial cells from CIA mice. VIP treatment *in vivo *was able to affect the transcriptional activity of both factors. Our data indicate that VIP is a viable candidate for the development of treatments for RA.

## Introduction

Rheumatoid arthritis (RA) is an autoimmune disease characterized by synovial inflammation, erosion of bone and cartilage, and severe joint pain [1-5]. Immunization of DBA-1 mice with type II collagen in complete Freund adjuvant induces the development of an inflammatory, erosive arthritis (collagen-induced arthritis (CIA) [6] accompanied by infiltration of the synovial membrane and synovial cavity as well as by extensive local bone and cartilage destruction and loss of bone mineral density [7]. This condition in mice mimics many of the clinical and pathological features of human RA. A link between the immune system and bone resorption is supported by the finding that several cytokines, such as tumor necrosis factor (TNF)α, IL-1β, IFNγ, IL-6, IL-11, and IL-17 with regulatory effects on immune function also contribute to bone homeostasis by enhancing bone resorption [8]. These cytokines have been identified in the rheumatoid synovium and could promote synovial membrane inflammation and osteocartilaginous resorption via stimulation of osteoclastic mediators [4,5,9,10].

A better understanding of the pathogenesis of bone erosion in RA relates to the discovery of osteoclast-mediated bone resorption that is regulated by the receptor activator of nuclear factor-κB (RANK) ligand (RANKL) [2-5,11,12]. RANKL is expressed by a variety of cell types involved in RA, including activated T cells and synoviocytes [8]. These cells, in the presence of cytokines like TNFα and macrophage colony stimulating factor, contribute to osteoclast differentiation and activation [8]. On the other hand, osteoprotegerin (OPG), which is a member of the TNF-receptor family expressed by osteoblasts, is a decoy receptor for RANKL [11,13]. OPG inhibits bone resorption and binds with strong affinity to its ligand, RANKL, thereby preventing RANKL binding to its receptor, RANK [11,13,14].

Vasoactive intestinal peptide (VIP) is a 28 amino acid peptide of the secretin/glucagon family present in the central and peripheral nervous system. It is also produced by endocrine and immune cells [15,16]. This peptide elicits a broad spectrum of biological functions, including anti-inflammatory and immunoregulatory properties, that lead to the amelioration or prevention of several inflammatory and autoimmune disorders in animal models and in human RA [17-23]. VIP has also been implicated in the neuro-osteogenic interactions in the skeleton. This function is supported by its presence in nerve fibers in the periosteum, the epiphyseal growth plate and the bone marrow [24]. The biological effects of VIP are mediated by G protein-coupled receptors (VPAC_1 _and VPAC_2_) that bind VIP and pituitary adenylate cyclase-activating polypeptide (PACAP) with equal affinity, and a PACAP selective receptor (PAC_1_) [25]. We have extensively studied the expression and distribution of these receptors in the immune system in cells of central and peripheral lymphoid organs [16-19]. Osteoclasts and osteoblasts have been shown to express different subtypes of VIP receptors [26,27]. The hypothesis that VIP may contribute to the regulation of osteoclast formation and activation has been investigated in different *in vitro *systems [28]. This study has shown a dual and opposite effect of VIP on osteoclast differentiation and activation [28]. Because bone resorption is a major pathological factor in arthritis and treatment with VIP significantly reduced the incidence and severity of arthritis in the CIA model [22], the aim of this study was to analyze the effects of VIP treatment *in vivo *on different mediators that interfere with bone homeostasis in this animal model.

## Materials and methods

### Animals

Male DBA/1J mice 6–10 weeks of age were purchased from The Jackson Laboratory (Bar Harbor, ME, USA). Water and food were provided *ad libitum *and all experiments were approved by the Institutional Animal Care and Use Committee of Complutense University in the Faculty of Biology.

### Induction, assessment and treatment of collagen-induced arthritis

Native bovine type II collagen (Sigma, St. Louis, MO, USA) was dissolved in 0.05 M acetic acid at 4°C overnight then emulsified with an equal volume of complete Freund adjuvant (DIFCO, Detroit, Michigan, USA). Mice were injected intradermally at the base of the tail with 0.15 ml of the emulsion containing 200 μg of type II collagen. At 21 days after primary immunization, mice were boosted intraperitoneally with 200 μg type II collagen in PBS. The analysis of mice was conducted every other day, with signs of arthritis onset monitored using paw swelling and clinical score as representative parameters. The study was conducted in a blinded manner by two independent examiners who determined the level of paw swelling by measuring the thickness of the affected hind paws with 0–10 mm callipers. Arthritis symptoms were assessed by using a scoring system (grade 0, no swelling; grade 1, slight swelling and erythema; grade 2, pronounced edema; grade 3, joint rigidity and ankylosis). Each limb was observed and graded with a maximum possible score of 12 per animal.

Three groups of animals were used in each experiment: control animals (no arthritic mice); a group of arthritic animals injected intraperitoneally with 1 nmol of VIP (Neosystem, Strasbourg, France) every other day between days 25 and 35 after primary immunization; and a group of arthritic mice injected with PBS instead of the VIP treatment.

### Histopathology

Thirty-five days after the first immunization, paws were fixed with 10% (w/v) paraformaldehyde, decalcified in 5% (v/v) formic acid, and embedded in paraffin. Sections (5 μm) were stained with hematoxylin-eosin-safranin O. Histopathological changes were scored in a blinded manner, using the following parameters. Cartilage destruction was graded on a scale of 0 to 3, from the appearance of dead chondrocytes (empty lacunae) to the complete loss of joint cartilage. Bone erosion was graded on a scale of 0 to 3, from normal appearance to completely eroded cortical bone structure.

### RNA extraction

Mice were sacrificed on day 35 after the first immunization and hind paws were homogenized using a tissue tearer. RNA was extracted using the Ultraspec phenol kit (Biotecx, Houston, TX, USA) as recommended by the manufacturer, resuspended in DEPC water and quantified by measuring the A260/280 nm.

### Quantitative real-time RT-PCR

Quantitative RT-PCR analysis was performed using the SYBR^® ^Green PCR Master Mix and RT-PCR kit (Applied Biosystems, Foster City, CA, USA) as suggested by the manufacturer. Briefly, reactions were performed in 20 μl with 20 ng RNA, 10 μl 2× SYBR Green PCR Master Mix, 6.25 U MultiScribe reverse transcriptase, 10 U RNase inhibitor and 0.1 μM primers. The sequences of primers used and accession numbers of the genes analyzed are summarized in Table 1. Amplification conditions were 30 minutes at 48°C, 10 minutes at 95°C, 40 cycles of denaturation at 95°C for 15 s, and annealing/extension at 60°C for 1 minute.

For relative quantification we used a method that compared the amount of target normalized to an endogenous reference. The formula used was 2^-ΔΔCt^, representing the *n*-fold differential expression of a specific gene in a treated sample compared with the control sample, where Ct is the mean of threshold cycle (at which the amplification of the PCR product is initially detected). ΔCt was the difference in the Ct values for the target gene and the reference gene, β-actin (in each sample assayed), and ΔΔCt represents the difference between the Ct from the control and each datum. Before using this method, we performed a validation experiment comparing the standard curve of the reference and the target to demonstrate that efficiencies were approximately equal [29]. The correct size of the amplified products was checked by electrophoresis.

### Cytokine determination in serum samples: ELISA assay

The amounts of IL-6, TNFα and IL-10 in serum were determined with a mouse capture ELISA assay. Briefly, a capture monoclonal anti-mouse IL-6, TNFα or IL-10 antibody (Pharmingen, Becton Dickinson Co, San Diego, USA) was used to coat micro titre plates (ELISA plates; Corning, NY, USA) at 2 μg/ml at 4°C for 16 h. After washing and blocking with PBS containing 3%(w/v) bovine serum albumin, serums were added to each well for 12 h at 4°C. Unbound material was washed off and a biotinylated monoclonal anti-human IL-6, TNFα or IL-10 antibody (Pharmingen, Becton Dickinson Co, San Diego, USA) was used at 2 μg/ml for 45 minutes. Bound antibody was detected by addition of avidin-peroxidase for 30 minutes followed by incubation of the ABTS substrate solution. Absorbance at 405 nm was measured 20 minutes after addition of substrate. A standard curve was constructed using various dilutions of mouse rIL-6, rTNFα or rIL-10 in PBS containing 10% (v/v) fetal bovine serum. The amounts of cytokine in the serum were determined by extrapolation of absorbance to the standard curve. The intra-assay and inter-assay variability for the determination was <5%. For IL-1β determination, murine IL-1β Quantikine^® ^M (R&D Systems, Minneapolis, USA) was employed according to the manufacturer's recommendations and absorbance was measured at 450 nm. For IL-4 determination, murine IL4 Eli-pair kit (Diaclone Research, Besancon, France) were used according to the manufacturer's recommendations and absorbance was measured at 450 nm.

### Determination of osteoprotegerin in serum

Mouse OPG in serum was assayed using a commercial murine OPG ELISA kit (mouse OPG/TNFSRSF11B immunoassay, R&D Systems). The standard curve was generated by serial dilution of a 2000 pg/ml stock provided by the manufacturer. Serum samples were diluted 1:5 with provided buffer and the assay was performed following the manufacturer's directions. Optical density was read at 450 nm with a reference filter set to 540 nm. The intra-assay variability was <5.5% and the limit of detection was 4.5 pg/ml.

### Electrophoretic mobility shift assays

Mice were sacrificed at day 35 after primary immunization, the rear limbs were removed, and the synovial membrane of the knee joints was carefully separated from the bone and cartilage by microscopic dissection. Cell suspensions were prepared by digestion of the synovial tissue in the presence of RPMI 1640, 250 mg/ml Colagenase D (Roche, Indianapolis, USA) and 0.1 mg/ml DNase I (Roche) for 2 h at 37°C, then samples were tapped through a 60 μm wire mesh. Nuclear extracts were prepared by the mini-extraction procedure of Schreiber *et al*. [30] with slight modifications. Briefly, 10^7 ^synovial cells centrifuged at 1,800 × g for 10 minutes. The cell pellets were homogenized with 0.4 ml of buffer A (10 mM HEPES pH 7.9, 10 mM KCl, 0.1 mM EDTA, 0.1 mM EGTA, 1 mM dithiothreitol (DTT), 0.5 mM phenylmethylsulphonylfluoride (PMSF), 10 μg/ml aprotinin, 10 μg/ml leupeptin, 10 μg/ml pepstatin, 1 mM NaN_3_, 5 mM NaF and 1 mM Na_3_VO_3_). After 15 minutes on ice, Nonidet P-40 was added to a final 0.5% concentration, the tubes were gently vortexed for 15 s and nuclei were sedimented and separated from cytosol by centrifugation at 12,000 × g for 40 s. Pelleted nuclei were washed once with 0.2 ml of ice-cold buffer A, and the soluble nuclear proteins were released by adding 0.1 ml of buffer C (20 mM HEPES pH 7.9, 0.4 M NaCl, 1 mM EDTA, 1 mM EGTA, 25% (w/v) glycerol, 1 mM DTT, 0.5 mM PMSF, 10 μg/ml aprotinin, 10 μg/ml leupeptin, 10 μg/ml pepstatin and 1 mM NaN_3_). After incubation for 30 minutes on ice, followed by centrifugation for 10 min at 12,000 × g at 4°C, the supernatants containing the nuclear proteins were harvested, the protein concentration was determined by the Bradford method, and aliquots were stored at -80°C for later use in EMSAs.

Double-stranded oligonucleotides (50 ng) corresponding to the NFκB and AP-1 sites (5'-AGTTGAGGGGACTTTCCCAGGC-3' and 5'-CGCTTGATGACTCAGCCGGAA-3', respectively), were end-labeled with γ^32^P-ATP (Amersham Pharmacia Biotech, NJ, USA) by using T4 polynucleotide kinase (Invitrogen, Carlsbad, CA, USA). For EMSAs with synovial cell nuclear extracts, 20,000 to 50,000 cpm of double-stranded oligonucleotides, corresponding to approximately 0.5 ng, were used for each reaction. The binding reaction mixtures (15 μl) were set up containing: 0.5 ng DNA probe, 8 μg nuclear extract, 2 μg poly(dI-dC)•poly(dI-dC) and binding buffer (50 mM NaCl, 0.2 mM EDTA, 0.5 mM DTT, 5% (w/v) glycerol and 10 mM Tris-HCl pH 7.5). The mixtures were incubated on ice for 15 minutes before adding the probe followed by another 20 minutes at room temperature, electrophoresed on a vertical 4% non-denaturing polyacrylamide gel using TGE buffer (50 mM Tris-HCl pH 7.5, 0.38 M glycine and 2 mM EDTA) and autoradiographed. For supershift assays, nuclear extracts were incubated for 15 minutes at room temperature with the specific antibody (1 μg of anti-p65, anti-p50, anti-cRel, anti-cFos, anti-cJun or anti-JunB) (Santa Cruz Biotechnology, Santa Cruz, CA, USA,) before the addition of the radiolabeled probe.

### Western blot analysis of IκB-α and phosphorylated cJun in cytoplasm extracts from synovial cells

For western blotting, the cytoplasm fraction (see above) containing 60 μg of protein were subjected to reducing SDS-PAGE (12.5%). After electrophoresis, the gel was electroblotted in Tris-glycine buffer containing 40% methanol onto a reinforced nitrocellulose membrane (Amersham). The membrane was blocked with TBS-T buffer (10 mM Tris, pH 8.0, 150 mM NaCl, 0.05% (w/v) Tween 20) containing 5% (v/v) milk powder for 1 h at room temperature, then incubated with primary antibodies at 1:500 dilutions, rabbit anti-mouse IgG against IκB-α (Santa Cruz) or with mouse IgG against phosphorylated-cJun (Santa Cruz), in TBS-T containing 1% (w/v) milk powder for 2 h at room temperature. The membrane was washed with TBS-T and incubated with secondary antibody: peroxidase-conjugated goat anti-rabbit IgG (Santa Cruz) or rat anti-mouse IgG (Santa Cruz) at 1:5000 dilutions for 1 h at room temperature. After washing three times in TBS-T for 5 minutes each, and once in TBS for 5 minutes, the membrane was drained quickly and subjected to the enhanced chemiluminiscence detection system (PIERCE). The X-ray films were exposed for 5 to 20 minutes.

### Statistical analysis

All data were expressed as mean ± SEM. Multiple-sample comparison (analysis of variance) was used to test differences between groups for significance. A value of p < 0.05 was considered to be significant. The program Statgraphics plus 5.0 (Statpoint Inc, Virginia, USA) was used for all statistical calculations.

## Results

### VIP modulates serum levels of cytokines implicated in bone homeostasis

We have previously reported the beneficial effects of VIP in a CIA model [22]. VIP improves clinical symptoms, decreasing the incidence and severity of CIA in mice. Notably, histopathological analysis of joints showed that inflammation, cartilage destruction and bone erosion were abrogated. A link between inflammation and bone homeostasis has been attributed to the effects of cytokines such as IL-1, TNFα, and IL-6 on bone resorption. Other cytokines, such as IL-4 and IL-10 have been shown to have protective effects if they are administered systemically [31]. We have previously reported that VIP treatment modulates the expression of different cytokines in the joints of CIA mice [22].

**Figure 1:**
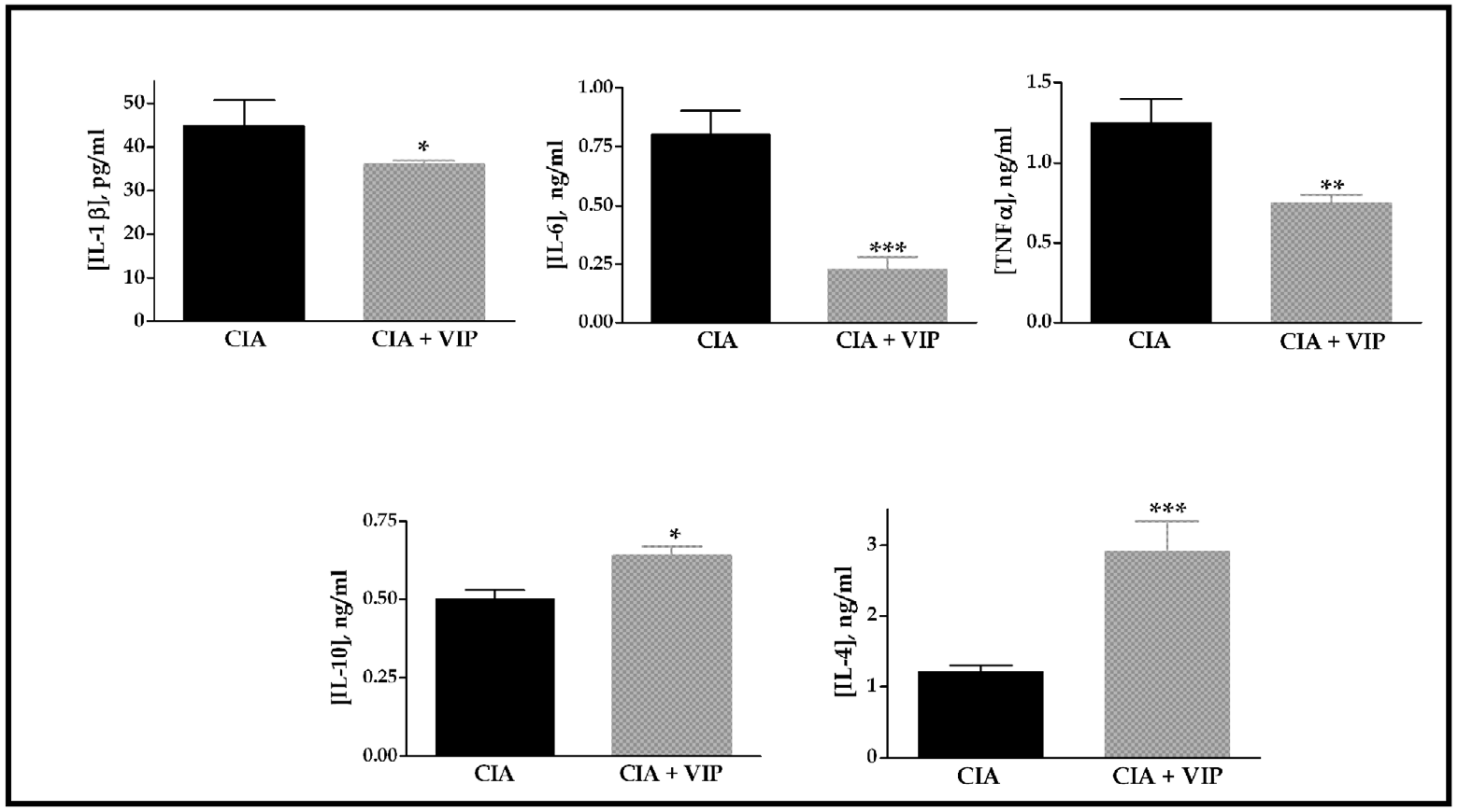
Cytokine circulating levels in mice at the end of treatment in the collagen-induced arthritis (CIA) model. IL-1β, tumor necrosis factor (TNF)α, IL-6, Il-10 or IL-4 were measured (mean ± SEM) by ELISA in arthritic animals and the same animals treated with VIP. On day 10 of VIP treatment, differences between the arthritic group and the CIA group treated with vasoactive intestinal peptide (VIP) were statistically significant (*p < 0.05, **p < 0.01, ***p < 0.001). Results are the mean ± SEM of two separate experiments with 10 animals per group.

Treatment of established CIA with VIP (1 nmol every other day per animal) resulted in suppression of disease activity (Table 2). Both cartilage pathology and bone destruction were reduced in VIP treated animals by the end of the experiment as revealed by histology. Furthermore, treatment reduced serum levels of IL-1β, TNFα, and IL-6, while circulating levels of IL-4 and IL-10 were higher in the VIP treated group (Fig. 1).

### Affect of VIP treatment on mRNA expression of inflammatory mediators and cytokines related to bone destruction

Bone degradation in the vehicle treated CIA group was seen as a reduction in the development of bone trabeculae and the presence of osteoclasts located at the sites of bone destruction. Osteoclasts implicated in bone resorption are controlled by an intricate interplay between several systemic factors and an array of local factors such as cytokines, inflammatory mediators and growth factors. As well as IL-1β, TNFα, and IL-6, local inflammatory mediators, such as prostaglandin E-2 (PGE-2), and nitric oxide (NO), as well as IL-11 and IL-17, have been shown to promote osteoclast differentiation and activation.

**Figure 2:**
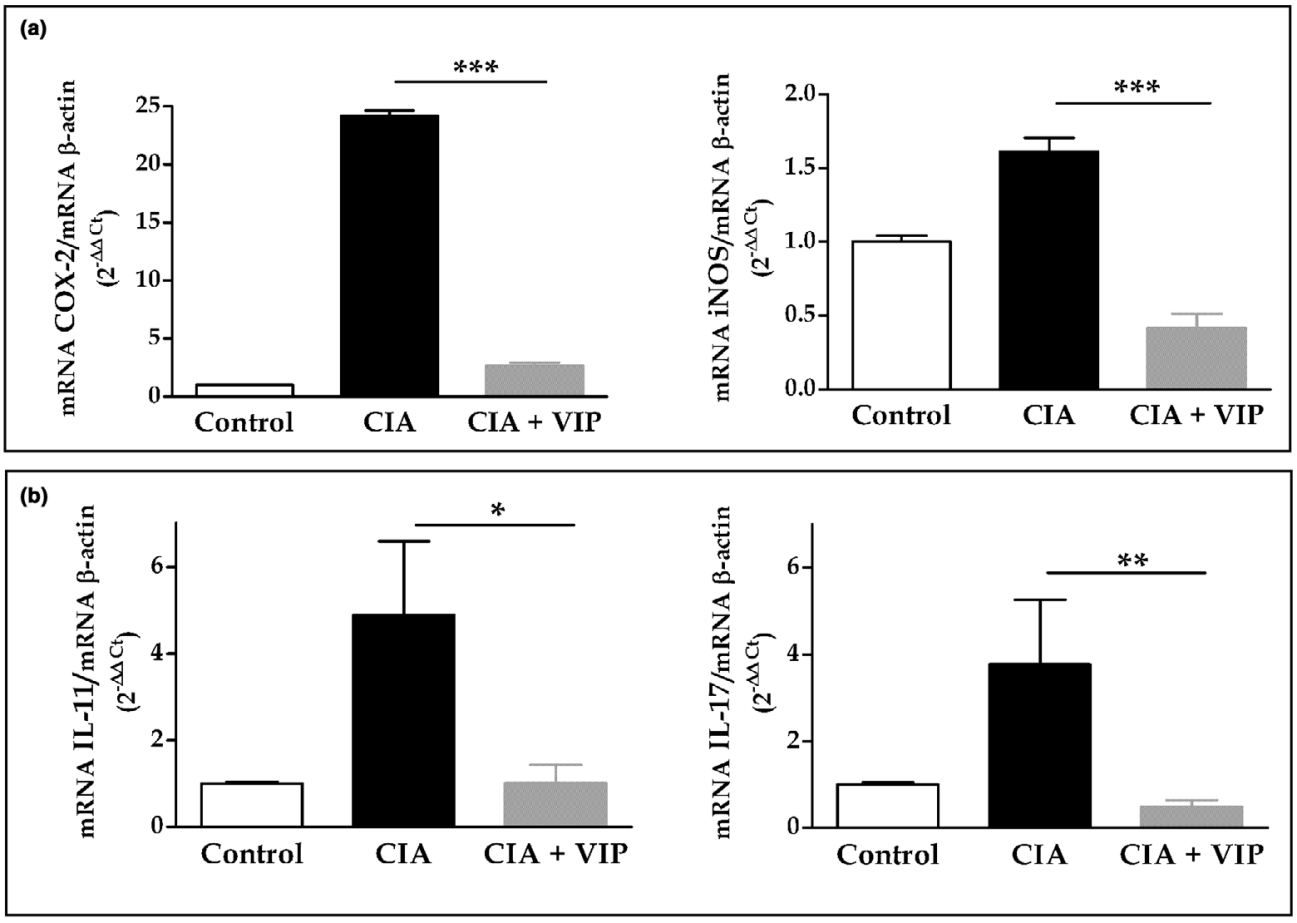
mRNA expression of inflammatory mediators and cytokines related to bone destruction. **(a) **Expression of mRNA for cyclooxygenase-2 (COX-2) and inducible nitric oxide synthase (iNOS) in the hind paws was measured by quantitative real-time PCR and corrected by mRNA expression for β-actin in each sample (see Materials and methods). **(b) **Expression of mRNA for IL-11 and IL-17 in the hind paws was measured by quantitative real-time PCR and corrected by mRNA expression for β-actin in each sample (see Materials and methods). On day 10 of vasoactive intestinal peptide (VIP) treatment, differences between the arthritic group and the CIA group treated with VIP were statistically significant (*p < 0.05, **p < 0.01, ***p < 0.001). Results are the mean ± SEM of two separate experiments with 10 animals per group.

To study the local expression of these factors we performed quantitative RT-PCR of the enzymes involved in the synthesis of these mediators (cyclooxygenase-2 (COX-2) and inducible nitric oxide synthase (iNOS)) as well as IL-11 and IL-17 in mRNA extracted from the joints. COX-2 and iNOS expression increased 25-fold and almost 2-fold, respectively, in the joints of CIA mice compared with the joints of control (non-CIA) mice (Fig. 2a). Also, IL-11 and IL-17 mRNA expression showed a four-fold increase in CIA mice (Fig. 2b). In CIA mice treated with VIP, the mRNA levels of COX-2, IL-11, and IL-17 in the joints were reduced compared with vehicle treated CIA mice, being similar to those of control (non-CIA) mice. The inhibition of iNOS expression was even higher.

### VIP modulates the RANK/RANKL/OPG system in the arthritic joint

**Figure 3:**
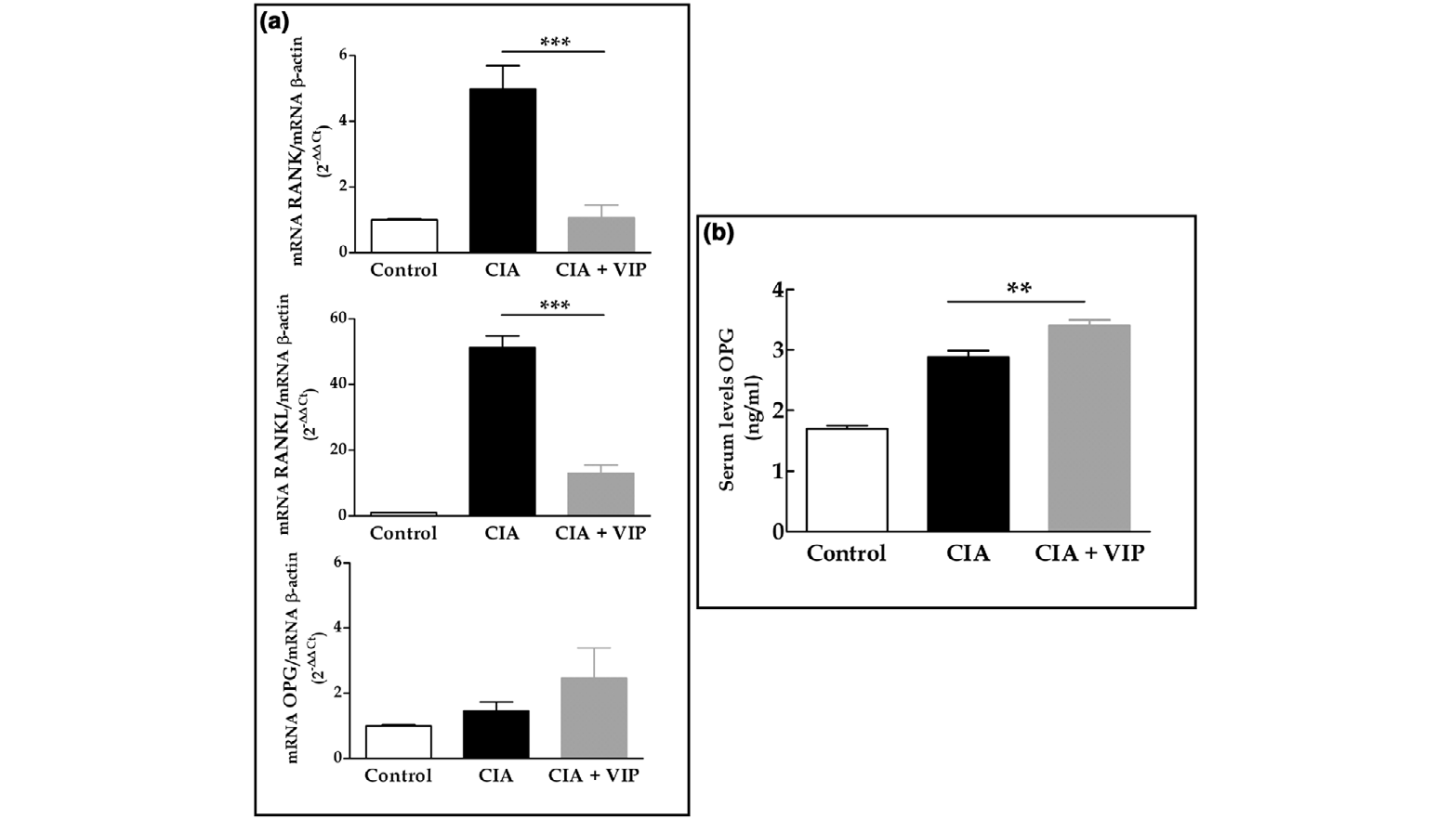
Vasoactive intestinal peptide (VIP) modulates the pattern of expression of the RANK/RANKL/OPG system in joints from mice with collagen-induced arthritis (CIA). **(a) **Expression of mRNA for receptor activator of nuclear factor-κB (RANK), receptor activator of nuclear factor-κB ligand (RANKL) or osteoprotegerin (OPG) in the hind paws was measured by quantitative real time PCR and corrected by mRNA expression for β-actin in each sample (see Materials and methods). **(b) **Serum levels of OPG in control, CIA or VIP-treated CIA mice were determined by ELISA. On day 10 of VIP treatment, differences between the arthritic group and the CIA group treated with VIP were statistically significant (**p < 0.01, ***p < 0.001). Results are the mean ± SEM of two independent experiments with 10 animals per group

As noted above, a link between the activation of the immune system and bone destruction is consistent with the finding that several cytokines contribute to bone resorption via stimulation of osteoclastic mediators. Mechanisms involved in this process operate by modulating the expression of RANK, RANKL and OPG. To study the modulation of the RANK/RANKL system and the ratio of RANKL to OPG by VIP during CIA development we performed quantitative RT-PCR in mRNA extracts from the joints of the different groups of animals. We also detected circulating OPG levels by ELISA in serum samples. The mRNA expression of RANK and RANKL was heavily stimulated in joints after CIA induction (Fig. 3a). In particular, CIA induction was accompanied by a 50-fold increase in RANKL expression in the affected joints. Though we also found a small increase in OPG mRNA in the same animals, no significant differences in OPG expression levels were detected after CIA induction. In spite of this small difference in its expression at the local level, however, the OPG circulating levels were significantly higher after CIA induction (Fig. 3b). On the other hand, the RANKL/OPG ratio was strongly enhanced in CIA mice (Table 3). VIP treatment of CIA mice resulted in a significant reduction in the expression of both RANK and RANKL, the mRNA levels of which in joints fell to near control values (non-CIA mice). Although in VIP treated mice OPG mRNA levels were slightly increased, a seven-fold drop in the RANKL/OPG ratio was observed (Table 3). The circulating levels of OPG were also significantly higher in VIP treated mice compared with CIA mice (Fig. 3b).

### VIP prevents *in vivo *NFκB translocation and inhibits c-Jun N-terminal kinase

**Figure 4:**
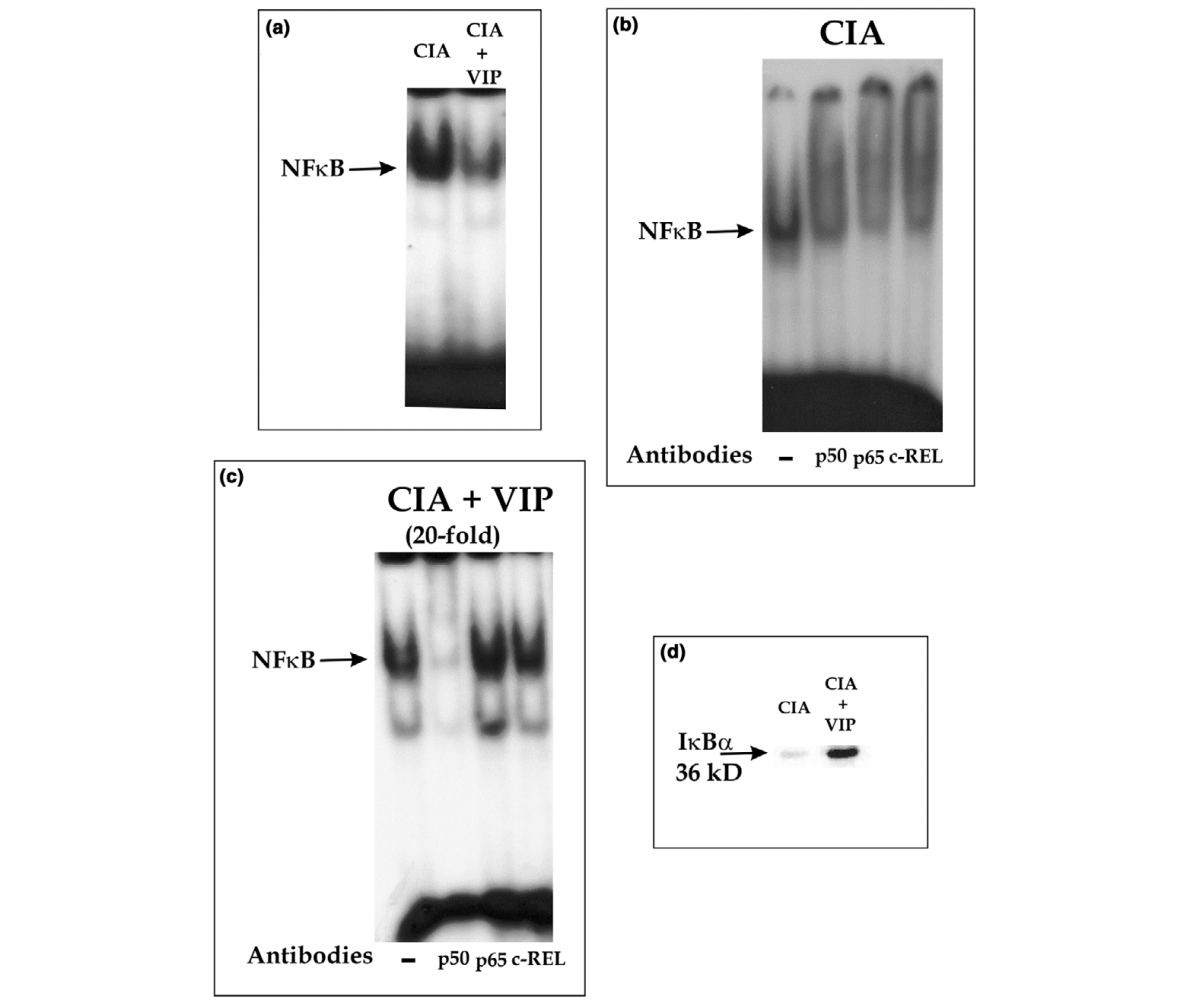
Effect of vasoactive intestinal peptide (VIP) on NFκB binding and IκB degradation in synovial cells from mice with collagen-induced arthritis (CIA). **(a) **EMSA results from nuclear extracts of synovial cells from CIA or VIP-treated CIA mice, using a radiolabeled oligonucleotide containing the NFκB consensus binding site. **(b) **Supershift assay on nuclear extracts of CIA mice using anti-p50, anti-p65 or anti-cRel. **(c) **Supershift assay (20-fold amplified) on nuclear extracts of VIP-treated CIA mice using anti-p50, anti-p65 or anti-cRel. **(d) **Western blot analysis showing immunoreactive IκBα (36 kDa) in cytoplasmic fractions of synovial cells from CIA and VIP-treated CIA mice. A representative experiment of three is shown.

Crucial events in signalling by RANKL and other osteoclastic cytokines are the translocation of NFκB to the nucleus and the activation of c-Jun N-terminal kinase (JNK), which leads to the activation of AP-1 [32,33]. A central role for these transcription factors is supported by the fact that both are activated by the tumor necrosis factor receptor-associated factor (TRAF) family of signal transducers and selective inhibition of NFκB blocks osteoclastogenesis and prevents inflammatory bone destruction *in vivo *[32,34]. Previous studies have shown that VIP induces a downregulation of NFκB transcriptional activity in human monocytes in culture [35,36], as well as an AP-1 binding decrease, and a marked change in the composition of the AP-1 complexes from c-Jun/c-Fos to JunB/c-Fos [36,37]. To investigate the molecular mechanism underlying the bone protective effect of VIP in CIA we studied the activities of NFκB and AP-1 in nuclear extracts of cell suspensions from joints by EMSA and in cytoplasmic extracts by western blotting. NFκB binding activity was greatly reduced in mice treated with VIP compared with vehicle treated CIA mice (Fig. 4a). Supershift experiments indicated that in vehicle treated CIA mice, the DNA protein complex appeared to contain p50, p65 and cRel (Fig. 4b); however, the residual binding activity detected in mice treated with VIP consisted of p50 homodimers (Fig. 4c). NFκB binding activity inhibition in VIP treated mice might be attributed to a reduction in IκBα phosphorylation degradation, since IκBα protein levels were increased in the cytoplasm as determined by western blot (Fig. 4d).

**Figure 5:**
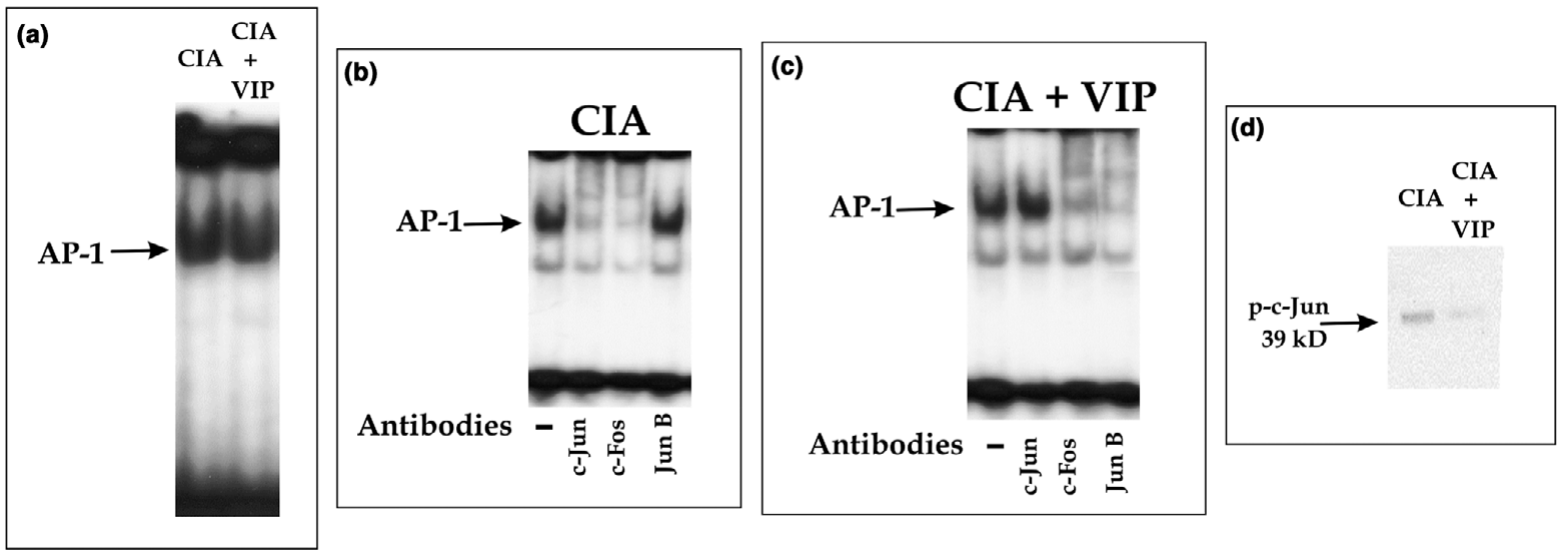
AP-1 binding and c-Jun activation in synovial cells from mice with collagen-induced arthritis (CIA) after vasoactive intestinal peptide (VIP) treatment. **(a) **EMSA results from nuclear extracts of synovial cells from CIA or VIP-treated CIA mice, using a radiolabeled oligonucleotide containing the AP-1 consensus binding site. **(b) **Supershift assay on nuclear extracts of CIA mice using anti-c-Jun, anti-c-Fos or anti-Jun B. **(c) **Supershift assay on nuclear extracts of VIP-treated CIA mice using anti-c-Jun, anti-c-Fos or anti-Jun B. **(d) **Western blot analysis showing immunoreactive phosphorylated c-Jun (39 kDa) in cytoplasmic fractions of synovial cells from CIA and VIP-treated CIA mice. A representative experiment of three is shown.

AP-1 DNA binding activity was higher in CIA mice and was not affected by VIP treatment, as determined by EMSA in nuclear extracts of cell suspensions from joints (Fig. 5a). Transcriptional activity of the AP-1 complex, however, is different in CIA mice and VIP treated animals. The supershift assay showed that the AP-1 complex in CIA is formed of transcriptionally active c-Jun/c-Fos heterodimers (Fig. 5b), while in VIP treated animals the AP-1 complex is formed by the transcriptionally inactive heterodimer c-Fos/Jun-B (Fig. 5c). The shift in the composition of the AP-1 complex may be mediated by inhibition of JNK activity because the western blot analysis indicated that phospho-c-Jun decreases in the cytoplasm after VIP treatment (Fig. 5d).

## Discussion

Data presented in this report indicate that VIP treatment prevents bone erosion in the CIA model of RA. Several mechanisms may account for this effect. VIP inhibits local and systemic levels of pro-inflammatory mediators implicated in bone resorption, such as IL-1β, IL-6, IL-11, IL-17, TNFα, PGE and NO, while the circulating levels of cytokines with bone protective effects, such as IL-4 and IL-10, are increased. On the other hand, VIP modulates the RANK/RANKL/OPG system, which is biased toward bone formation. Finally, osteoclast function may be inhibited as it depends on NFkB and AP-1 transcription factor activity, which is impaired in VIP treated mice.

VIP has been shown to regulate several bone cell functions; it affects bone resorbing activity of isolated osteoclasts and osteoclast formation [28] as well as osteoblast anabolic processes [24]. These effects are mediated by the presence of different VIP receptors in both types of bone cells: VPAC_1 _and PAC_1 _have been detected in osteoclasts [26] while VPAC_2 _is expressed in osteoblasts and VPAC_1 _is induced in advanced cultures of this cell type [27]. *In vitro *studies with isolated cells have shown contradictory results; while VIP has been shown to promote the formation of mineralised nodules in cultures of osteoblasts [24], it induces a transient inhibition and a delayed stimulation of osteoclast activity [38]. Our results show that VIP treatment *in vivo *in pathological conditions such as RA results in the prevention of bone destruction.

Cytokine balance contributes to the onset and progression of inflammation and skeletal destruction during RA. In this respect, TNFα, IL-1β and IL-6 have been shown to be dominant in the induction of inflammation and bone erosion [39-41], while IL-4 and IL-10 have potent anti-inflammatory effects and suppress cartilage and bone pathology in RA [31]. Both a systemic and a paracrine mode of action can be postulated for these agents. Alteration of the systemic balance of cytokines has been studied by blocking TNFα and IL-1β using biological agents such as anti-TNFα or IL-1 inhibitors [39]. Therefore, a combined cytokine and anti-cytokine therapy has been proposed as being the more effective for achieving an anti-inflammatory and anti-destructive therapy for RA. VIP thus emerges as a new, promising biological agent in this sense, as treatment of CIA mice with this peptide shifts the systemic balance of cytokines toward a bone protecting pattern that acts to both lower serum levels of TNFα, IL-1β and IL-6 and raise the levels of IL-4 and IL-10, as described in this report.

Bone loss in RA is indirectly mediated mainly by cytokines produced by macrophages, fibroblasts and T cells of the synovial tissue. These cytokines lead to the differentiation of osteoclast precursors and activate osteoclasts. Macrophage and fibroblast derived inflammatory cytokines such as IL-1β and TNFα perpetuate inflammation in a paracrine manner. In a previous report, we have shown that VIP reduces the expression of such mediators in the joint microenvironment of arthritic mice [22]. At the same time, VIP augments the local production of the anti-inflammatory cytokine IL-10 and the IL-1 inhibitor IL-1Ra [22]. PGE [42] and NO [43] are two potent mediators induced by inflammatory cytokines that stimulate their osteoclastogic activities. They are also inhibited in the joints of VIP treated mice, as can be deduced from the lower expression of iNOS and COX-2.

VIP can also impair osteoclast differentiation in RA through its effect on T cell differentiation and activation. T cells present in the synovial tissue in RA express a Th1/Th0 pattern of cytokine secretion [44]. Activated T cells and T cells from RA synovial tissue express both the membrane-bound and soluble forms of RANKL, which induce the differentiation of osteoclast precursors [45]. Cytokines also participate in this process. IL-17 is a cytokine produced by a subset of activated memory Th1/Th0 cells [46] that has been shown to be an important osteoclast differentiation factor, inducing RANKL expression leading to bone erosion in arthritis [10]. IL-11 also supports osteoclast formation by increasing RANKL expression in a STAT (Signal transducers and activators of transcription) activation dependent mechanism [47]. As we have described in this report, VIP treatment greatly reduces the local expression of both these cytokines in the joints of arthritic mice, which may account for the block in joint erosion induced in the CIA model. Additionally, VIP shifts the immune response towards a Th2 pattern of cytokine secretion [17], which inhibits the production of inflammatory and Th1 cytokines [48].

Most of the osteoclastogenic factors present in RA joints are thought to act indirectly, enhancing RANKL expression and thereby altering the RANK/RANKL/OPG system, which is the final regulator of bone resorption [2,3,49]. RANK is expressed on the surface of haematopoietic osteoclast progenitors that belong to the monocyte/macrophage lineage, and also on mature osteoclasts, as well as on T cells and dendritic cells. In arthritis, osteoclast precursors that express RANK recognize RANKL through cell-to-cell interaction with osteoblasts/stromal cells, and differentiate into osteoclasts [50]. In the present study, we report a high level of RANK expression in the joints of arthritic mice, probably induced by the recruitment of osteoclast precursors induced by the local production of chemokines chemotactic for monocytes [51]. We also describe how VIP lowers the expression of RANK in the joints of CIA mice to the levels detected in non-arthritic control mice. This effect may be due to the inhibition of RANK synthesis or, alternatively, to the inhibition of monocyte recruitment; we have reported previously that VIP inhibits the local expression of the monocyte chemoatractant chemokines CCL3 (MIP1α) and CCL2 (MCP-1) [22,23]. RANKL expression can be upregulated by bone resorbing factors such as glucocorticoids, vitamin D, IL-1β, IL-6, IL-11, IL-17, TNFα, PGE_2_, or parathyroid hormone in osteoblasts. RANKL is expressed on the cell surface of activated T cells and can be detected in both synovial cells and infiltrating cells by *in situ *hybridization at the onset of clinical signs of arthritis in animal models [52]. T-cell activation in RA patients may lead to osteoclastogenesis within the synovium, probably via RANKL secretion by activated T cells in an environment conducive to osteoclast differentiation from synovial macrophages. This mechanism may contribute to the bone destruction seen in RA [14].

VIP has been reported to inhibit the expression of RANKL and RANK induced by vitamin D in mouse bone marrow cultures [28]. Results shown in this report indicate that VIP reduces the expression of RANK and RANKL in the joints of arthritic mice, and may account for the bone protective properties of VIP in RA. On the other hand, its effects on the expression of OPG further support the postulated bone protective property of VIP. This molecule is secreted by stromal cells and osteoblasts and competitively inhibits RANKL binding to RANK on the cell surface of osteoclast precursor cells and mature osteoclasts, thus inhibiting the osteoclastogenic actions of RANKL. Excessive production of RANKL and/or a deficiency of OPG could, therefore, contribute to the increased bone resorption typified by the focal bone erosion and bone loss in RA. Our data indicate that OPG circulating levels rise in CIA, as has been reported during inflammation [14]. These levels were even higher in VIP treated mice. In this way, the ratio of RANKL-RANK to OPG that determines the erosive nature of RA is greatly reduced by VIP, accounting for the bone protection achieved by the treatment.

The molecular mechanisms underlying the discussed effects of VIP in bone protection during RA (mainly cytokine secretion, RANKL expression, and osteoclast differentiation) may involve the transcription factors NFκB and AP-1. Several cell types share these signalling pathways to express mediators implicated in tissue damage and destruction. After exposure to pro-inflammatory cytokines, the IκB kinase (IKK) signal complex is activated in synoviocytes, leading to phosphorylation of IκB. We describe in this report that IκB phosphorylation is inhibited in the arthritic joints of mice treated with VIP. NFκB is activated in this manner in the synovium of patients with RA and regulates genes encoding proteins that contribute to inflammation, including inflammatory cytokines such as TNFα, IL-1β, IL-6 and chemokines as well as enzymes such as iNOS and COX-2. NFκB is also crucial for the differentiation of osteoclasts and its selective inhibition blocks RANKL induced osteoclastogenesis both *in vitro *and *in vivo *[32]. The MAPK (Mitogen-activated protein kinases) pathway is also involved and particularly the JNK pathway, which has been implicated in the regulation of matrix metalloproteinases. As reported here, JNK activity in the joints of arthritic mice is affected by VIP treatment. Our understanding of the signal transduction pathways implicated in RA has led to drug development programmes targeting MAPK and NFκB inhibitors [53]. Several of these compounds, however, have been shown to be toxic. VIP on the other hand has been shown to target these signalling pathways and no toxicity has been cited for this peptide. Ourselves and others have previously reported that VIP inhibits the nuclear translocation of NFκB and also the JNK signalling pathway in LPS (lipopolysaccharide) stimulated macrophage and monocytic cell lines [35-37] In the present report, we describe that this mechanism also operates *in vivo *and may involve other cell types involved in the pathogenesis of RA.

In summary, the protective effect of VIP in bone destruction during CIA could be due to different mechanisms that are not mutually exclusive. One would be an indirect mechanism that works via decreasing proinflammatory cytokines and other mediators involved in the differentiation and activation of osteoclast-precursor cells, and increasing anti-inflammatory cytokines. A second would be the VIP-induced modification of the cell types present in the joint, which would decrease the amount of Th1-lymphocytes that express RANKL. And a third would be a VIP-induced direct effect on OPG, RANK or RANKL expression on skeletal tissue, fibroblast or immune cells present in the inflamed joint.

## Conclusion

We have shown that VIP treatment in CIA mice reduces the local and systemic levels of osteoclastogenic mediators, such as TNFα, IL-1β, IL-6, IL-11, IL-17, PGE and NO. This reduction is accompanied by a large decrease in the RANK-RANKL/OPG ratio. Molecular mechanisms associated with these events include a reduction in the activity of the transcription factor NFκB and a change in the activity of AP-1. Our results highlight the possibility of the therapeutic application of VIP in the treatment of human RA.

## Abbreviations

CIA = collagen-induced arthritis; COX-2 = cyclooxygenase-2; DTT = dithiothreitol; ELISA = enzyme-linked immunosorbent assay; EMSA = electrophoretic mobility shift assay; IFN = interferon; IL = interleukin; iNOS = inducible nitric oxide synthase; JNK = c-Jun N-terminal kinase; NO = nitric oxide; OPG = osteoprotegerin; PAC_1 _= PACAP receptor; PACAP = pituitary adenylate cyclase-activating polypeptide; PBS = phosphate-buffered saline; PGE-2 = prostaglandin E-2; PMSF = phenylmethylsulphonylfluoride; RA = rheumatoid arthritis; RANK = receptor activator of nuclear factor-κB; RANKL = receptor activator of nuclear factor-κB ligand; TNF = tumor necrosis factor; VIP = vasoactive intestinal peptide; VPAC_1 _= type 1 VIP receptor; VPAC_2 _= type 2 VIP receptor.

## Competing interests

We have signed a research agreement with a company ((Genetrix S.L., Spain)) interested in the development of new therapeutic approaches to treat RA, although this company did not finance this manuscript. We have no stocks or shares with any organization. We do not have any patent application related to the content of this manuscript. We do not have any financial or non-financial competing interest.

## Authors' contributions

YJ made substantial contributions to the conception and design of this study and the acquisition, analysis and interpretation of data. CA carried out the histopathological studies. CM prepared the samples and gave final approval of the manuscript for publication. AA was involved in the real-time analysis. IGC prepared the samples and performed the statistical analysis. FR was involved in the design of the figures. RPG made substantial contributions to the conception and design of the study, gave final approval of the manuscript for publication. JL made substantial contributions to the conception and design of the study and was involved in revising the article critically for important intellectual content. All authors read and approved the final manuscript.

## Figures and Tables

**Table 1:** Primer sequences for several factors involved in bone regulation and for β-actin

Gene name	Genebank accession number	Sequence position	Primers	Sequence
β-Actin	NM007393	694–831	Bactin.for Bactin.rev	5'-AGAGGGAAATCGTGCGTGAC-3' 5'-CAATAGTGATGACCTGGCCGT-3'
IL-11	NM008350	350–450	IL-11.for IL-11.rev	5'-TGATGTCCTACCTCCGGCAT-3' 5'-TTCCAGTCGGGCTTGCAG-3'
IL-17	NM010552	146–246	IL-17.for IL-17.rev	5'-CCTCAAAGCTCAGCGTGTCC-3' 5'-GAGCTCACTTTTGCGCCAAG-3'
COX-2	NM011198	854–954	COX-2.for COX-2.rev	5'-GGTGGAGAGGTGTATCCCCC-3' 5'-ACTTCCTGCCCCACAGCA-3'
iNOS	NM010927	872–972	iNOS.for iNOS.rev	5'-AACAATGGCAACATCAGGTCG-3' 5'-CCAGCGTACCGGATGAGCT-3'
OPG	U94331	831–931	OPG.for OPG.rev	5'-AGAGCAAACCTTCCAGCTGC-3' 5'-CGCTGCTTTCACAGAGGTCA-3'
RANK	AF19046	1422–1440	RANK.for RANK.rev	5'-TGCCTACAGCATGGGCTTT-3' 5'AGAGATGAACGTGGAGTTACTGTTT3'
RANKL	AF53713	606–680	RANKL.for RANKL.rev	5'-TGGAAGGCTCATGGTTGGAT-3' 5'-CATTGATGGTGAGGTGTGCAA-3'

COX-2, cyclooxygenase-2; iNOS, inducible nitric oxide synthase; OPG, osteoprotegerin; RANK, receptor activator of nuclear factor-κB; RANKL, receptor activator of nuclear factor-κB ligand.

**Table 2:** Effect of VIP treatment of mice with collagen-induced arthritis

	Clinical score	Cartilage destruction	Bone erosion
CIA	5.08 ± 0.24	2.8 ± 0.07	2.13 ± 0.12
CIA + VIP	1.56 ± 0.17^a^	0.61 ± 0.22^a^	0.25 ± 0.11^a^

Clinical score (mean ± SEM) was assessed on a scale of 0 to 6. Cartilage destruction and bone erosion (mean ± SEM) was graded on a scale from 0 to 3. On day 10 of vasoactive intestinal peptide (VIP) treatment, differences between the arthritic group and the collagen-induced arthritis (CIA) group treated with VIP were statistically significant (^a^p < 0.001).

**Table 3:** Ratio of RANKL to OPG in mice with collagen-induced arthritis

	CIA	CIA + VIP
RANKL	51.20 ± 3.57	13.08 ± 2.45^a^
OPG	1.46 ± 0.27	2.46 ± 0.92
RANKL/OPG	35.09 ± 3.18	5.30 ± 0.95^a^

The mRNA expression for RANKL and OPG in hind paws of mice with collagen-induced arthritis (CIA) was measured by quantitative real time PCR and corrected by mRNA expression for β-actin in each sample. On day 10 of vasoactive intestinal peptide (VIP) treatment, differences between the arthritic group and the CIA group treated with VIP were statistically significant (^a^p < 0.001). Results are the mean ± SEM of two independent experiments with 10 animals per group. OPG, osteoprotegerin; RANKL, receptor activator of nuclear factor-κB ligand.
